# The Internal Structural Dynamics of Elastin-Like Polypeptide Assemblies by ^13^C-Direct Detected NMR Spectroscopy

**DOI:** 10.1021/acs.analchem.4c05163

**Published:** 2025-02-17

**Authors:** Dörte Brandis, Pavel Kadeřávek, Dennis Kurzbach

**Affiliations:** † Institute of Biological Chemistry, Faculty of Chemistry, University of Vienna, Währinger Str. 38, 1090 Vienna, Austria; ‡ Vienna Doctoral School in Chemistry (DoSChem), University of Vienna, Währinger Str. 42, 1090 Vienna, Austria; § Central European Institute of Technology (CEITEC), Masaryk University, Kamenice 5, 625 00 Brno, Czech Republic; ∥ National Centre for Biomolecular Research (NCBR), Faculty of Science, Masaryk University, Kamenice 5, 625 00 Brno, Czech Republic

## Abstract

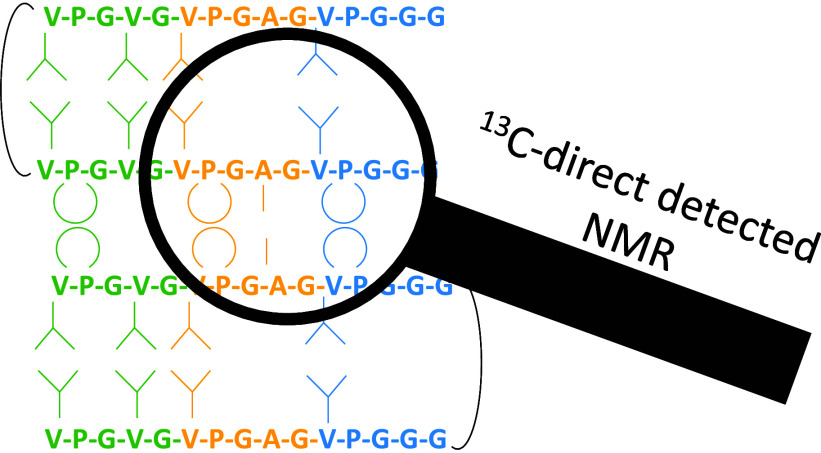

Elastin-like polypeptides (ELPs) are biocompatible polymers exhibiting lower critical solution temperature (LCST) behavior, making them valuable in various applications, including drug delivery and tissue engineering. This study addresses the atomistic-level understanding of ELP self-assembly, focusing on their internal structural dynamics. Conventional proton-detected nuclear magnetic resonance (NMR) spectroscopy faces limitations in studying ELP aggregates due to accelerated proton exchange processes, which cause significant resonance broadening. Herein, we show how to overcome this hurdle by using carbon-13-detected NMR. This method mitigates issues related to amide proton exchange, allowing for a residue-resolved view of the internal configuration of ELP aggregates. With this method, we record residue-resolved ^15^N relaxation rates, revealing three features. (i) Proline residues within the PGXGV pentapeptide repeats (X being any amino acid except proline) of ELP become motional restricted upon aggregation, indicating their role as interchain contacts. (ii) Pentapeptides with alanine guest residue X display particularly significantly reduced motional freedom upon aggregation. (iii) Even within large ELP aggregates, fast internal dynamics characterize the peptide chains in a way that is reminiscent of condensed liquid phases. The presented study is the first proof of concept that ^13^C-direct detection is a viable tool to delineate the internal structural dynamics of condensed ELP phases by NMR. It might, thus, help to foster new investigations of their aggregation mechanisms.

## Introduction

Elastin-like polypeptides (ELP) are biomimetic polymers that consist of PGXGV pentapeptide repeats (X being any guest residue except proline), which exhibit a lower critical solution temperature (LCST) behavior.^1−6^ LCST defines the temperature below which the components of a mixture are completely miscible.^7^ In the case of an ELP in aqueous solutions, this means that they lose their hydration shell once experiencing temperatures above their LCST, leading to reversible peptide aggregation and separation of condensed phases.^1−3,5,8−21^ This property renders them highly interesting as agents in applications ranging from protein purification,^3,22^ drug delivery,^20^ tissue engineering,^23^ and immunoassays^24^ to molecular actuation.^25^

Despite extensive research efforts, a detailed understanding of the molecular determinants that drive the LCST behavior is still lacking - a significant shortcoming considering that such knowledge is crucial for the rational design of ELP and their applications. However, the complexity of the LCST behavior, which is influenced by various solution conditions such as ionic strengths, peptide concentrations, hydration states, and pH, renders the detection of structure–function relationships very challenging.^9,21,26^ Additionally, the scarcity of methods to access the structural dynamics of ELP at high resolution further complicates the research. For instance, nuclear magnetic resonance (NMR) spectroscopy, a most widely used tool in solution-state structure determination, cannot readily be applied to ELP aggregates above the LCST due to heterogeneous dynamics and unfavorable exchange kinetics,^27^ leading to resonance broadening beyond the detection threshold. Only in exceptional cases, such as very short peptides, is NMR a viable tool for ELP structural analysis.^14,27−33^ This limitation hampers the assessment of the very informative NMR-derived residue-resolved chemical shifts and relaxation rates, which report on the structure and dynamics of proteins and peptides at residue resolution.^34^

Herein, we show that this problem can be overcome using ^13^C’-direct detected NMR,^28^ providing a very versatile tool that can readily enlighten the conformational dynamics within ELP aggregates at residue resolution. This method has the advantage that chemical and conformational exchange involving amide H^N^ protons (for ELP, most importantly, during the formation/dissociation of β-strands or turns)^35^ does not broaden the ^13^C’ resonances. Hence, ^1^H NMR signals lost upon ELP aggregation can be recovered using ^13^C-direct detection. Besides, the activity of the proline residues, which is essential for understanding ELPs, can be assessed with the herein-reported approach.

Hence, in comparison to the conventionally detected ^1^H-^15^N resonances, which are mostly limited to solute monomers, ^13^C-direct detection allows a glimpse into ELP aggregates.

It should be noted that heteronuclear-detected NMR of polymers has a long-standing history; in particular, the detection of ^13^C-relaxation rates has already been shown to be very useful in the analysis of ELPs. For example, differential relaxation rates,^36,37^ chain motions,^38^ and diffusion coefficients^39^ were employed to characterize the coexisting dilute and condensed phases. Herein, we capitalize on this body of work and include multidimensional, heteronuclear detection schemes for ^13^C’ NMR-based analysis of polymers and ELPs. This addition provides increased resolution and, at the same time, allows the assessment of the properties of adjacent nuclei, such as the amide ^15^N resonances in ELPs.

This methodological advancement is particularly important considering the recent considerable disagreement about the mechanism of ELP aggregation. In particular, it was discussed whether β-turn and β-sheet structures are present in ELP aggregates^23,25,40,41^ and/or monomeric species below the LCST.^30^ Further, the question of whether potential β-sheet-like structures are involved in the intermolecular contact between ELP chains remains to be answered.^26^

With the proposed method, we show that such answers come into reach. In particular, we report three new pieces of information about the internal configuration of ELP aggregates. First, turns with exposed proline residues become motionally severely restricted upon surpassing the LCST, suggesting their involvement in the interchain contacts. Second, we find that glycines in pentapeptides that have an alanine as guest residue become counterintuitively more confined than glycines in other repeat units. Third, the internal structural dynamics of the polypeptide chain within the aggregates are relatively fast, reminiscent of motions reported for condensed liquid phases of intrinsically disordered proteins.

## Methods and Materials

### Peptide Expression and Purification

The ELP-90 (see Figure S1 for the sequence) was expressed as an R5-fusion construct, connected by a TEV-cleavage site, was cloned into a pET-21a(+) expression vector and transformed into *E. coli* Rosetta2 cells. The R5 tag is not important in the current methodological study, but it is used for applications in other contexts. For protein expression, cells were incubated in 200 mL 2xYT medium at 37 °C overnight. The next day, the culture was diluted in 2 L 2xYT medium to reach an optical density of A600 = 0.3. Subsequently, the cells were cultured at 37 °C until an optical density of A600 = 0.6. Then, the cells were transferred to an M9 minimal Medium containing ^13^C_6_ glucose and ^15^N ammonium chloride for ^13^C and ^15^N labeling. The expression was induced with isopropyl-β-d-thiogalactopyranoside, and the culture was incubated at 30 °C overnight. After separation by centrifugation, cells were resuspended in a buffer consisting of 25 mM TRIS, 100 mM NaCl, and 2 mM β-mercaptoethanol at pH 8 and lysed by sonication. The soluble part was purified by Inverse Transition Cycling (ITC).^4^ For one cycle, the NaCl concentration of the solution was increased to 2 M, followed by heating it to 70 °C for 20 min, followed by rapid centrifugation at room temperature. The pellet was then resuspended on ice in a cold buffer, and the remaining protein precipitate was removed by centrifugation at 4 °C. After three cycles, SDS PAGE and mass spectrometry confirmed the purity of the product. The buffer was exchanged to 50 mM MES Buffer with 0.5 M NaCl at pH = 6.5 with an Amicon Ultra-15, with a10 kDa cutoff.

### NMR Spectroscopy

All NMR Experiments have been performed with an ELP concentration of 1 mM in 50 mM MES pH = 6.5 and 0.5 M NaCl in 9:1 H_2_O/D_2_O. All samples were measured in 5 mm OD Shigemi tubes. The final sample volume was 350 μL.

All 2D spectra were acquired on a Bruker NEO 600 MHz spectrometer equipped with a cryogenically cooled Prodigy TCI probe. 3D spectra for the signal assignment were recorded on a 950 MHz Bruker NEO spectrometer equipped with a helium-cooled TCI probe.

A combination of HNCO, HNCACB, and HNN spectra was used to assign the backbone resonances. HNCO, HNN, and HNCACB were recorded by using the pulse programs ’hncogpwg3d, ’best_hnngpwg3d’, and ’hncacbgp3d’ of the Bruker TopSpin 4 pulse program catalog. HNCO was recorded with spectral width and offset frequencies of 13157.895 Hz and 4.7 ppm for ^1^H, 2000.000 Hz, and 117.0 ppm for ^15^N and 2000.000 Hz and 171.993 ppm for ^13^C. HNN^42^ was recorded with spectral widths and offset frequencies 6250.000 Hz and 4.669 ppm for ^1^H and 1824.689 Hz and 122.000 ppm for ^15^N. HNCACB was recorded with spectral width and offset frequencies of 13157.895 Hz/4.7 ppm for ^1^H, 1926.228 Hz/117.0 ppm for ^15^N, and 13382.936 Hz/43 ppm for ^13^C.

Hydrogen-detected, ^15^N relaxation rates were recorded by ^1^H-^15^N correlation spectra using the pulse programs ‘hsqct1etf3gpsi3d’ and ‘hsqct2etf3gpsi3d’ with spectral widths and offset frequencies of 9615.385 Hz/4.7 ppm for ^1^H and 2128.799 Hz/117.0 ppm for ^15^N. The spectra were processed with NMRpipe^43^ and analyzed with SPARKY.^44^ For the obtained data and the recorded relaxation delays, see the Supporting Information.

NOESY was recorded using the noesygpphw5 pulse sequence of the Bruker TopSpin 4 pulse sequence catalog. The spectral width was 9.8 ppm in both dimensions, and the carrier frequency was set to 4.7 ppm.

DOSY spectra were recorded on a 950 MHz Bruker NEO spectrometer using the ‘stebpgp1s19′ pulse program with 16 data points for ^1^H and ^13^C in a linear variation of the z-gradient strength from 0 to 16 (^1^H) or 140 (^13^C) T^2^m^–2^. The diffusion delay was chosen to 20 ms, and data was analyzed using GNAT,^44^ by integrating peaks of the methyl groups and fitting the integral vs gradient strength. Hydrodynamic radii were extracted using the Stokes–Einstein law, assuming a spherical model.^45−47^ 1,4-Dioxane was used for internal referencing.

Carbon-detected, ^15^N relaxation rates were recorded by ^13^C-^15^N correlation spectra with various delays and pulse programs reported in references^52−56^ with spectral widths and offset frequencies of 4545.450 Hz/174.000 ppm for ^13^C and 1824.688 Hz/122.0 ppm for ^15^N. The data were processed with Topspin 4.2 and analyzed with CcpNmr Analysis 2.5.2.^48^ Data were zero-filled to four times the number of points and apodized using a 180° shifted sine-bell squared window function prior to Fourier transformation. In all cases, a polynomial function of the fifth degree achieved baseline correction.

Relax-EXSY was recorded at 35 °C using the same pulse sequences again and fitted as detailed in reference.^49^ The longitudinal relaxation rates were recorded using D_2_O concentrations of 9%, 30%, and 50%.

Note that the phase transitions of ELP often span >10 K between states of full aggregation and complete dissolution on the molecular scale.^50^ Hence, to make sure that no partially aggregated states influenced our analysis, we chose a large temperature difference of 20 °C between 15 and 35 °C for our experiments.

## Results and Discussion

The inapplicability of biomolecular NMR spectroscopy to ELP aggregates can primarily be traced back to the fast chemical and/or conformational exchange processes within these self-assemblies.^51−54^ These cause resonance line broadening, which typically results in the suppression of signals stemming from residues within the aggregates. Herein, we capitalize on the fact that this problem can effectively be mitigated by means of heteronuclear ^13^C-detection schemes.

As will be shown below, mainly chemical exchange processes involving the amide H^N^ proton effectively contribute to the transverse relaxation rate constant *R*_2_ in conventional proton-detection schemes of ELP aggregates. However, using ^13^C-detection, such processes are canceled. In other words, hydrogen bond formation and dissociation processes, as well as chemical proton exchange, cannot broaden the detected resonance when considering the effects of isotope shifts as negligible at low and constant solvent deuteration levels. This feature is particularly valuable for NMR of peptide aggregates in which hydrogen bond formation rates can become very fast,^55^ e.g., due to the formation and dissociation of transient β-turn and β-sheet structures in the case of ELP.^56^

Hence, when aiming to assess structural dynamics via the typically recorded amide ^15^N relaxation rates of the peptide backbone, it is beneficial to detect these through correlations with the neighboring carbonyl ^13^C’ nucleus instead of H^N^.

With this information in mind, we investigated a 450-amino-acid-long ELP. We chose a widely employed variant^4^ to make the results as representative as possible. This ELP consists of 90 repeats with 27 G, 18 A, and 45 V residues as the X in PGXGV (for the complete primary sequence, see the Supporting Information) and is routinely used as a protein purification tag.^4^ At temperatures above 35 °C, this ELP aggregates as its LCST is surpassed (in a 0.5 M NaCl buffer; Supporting Information Figure S1).

**Figure 1:**
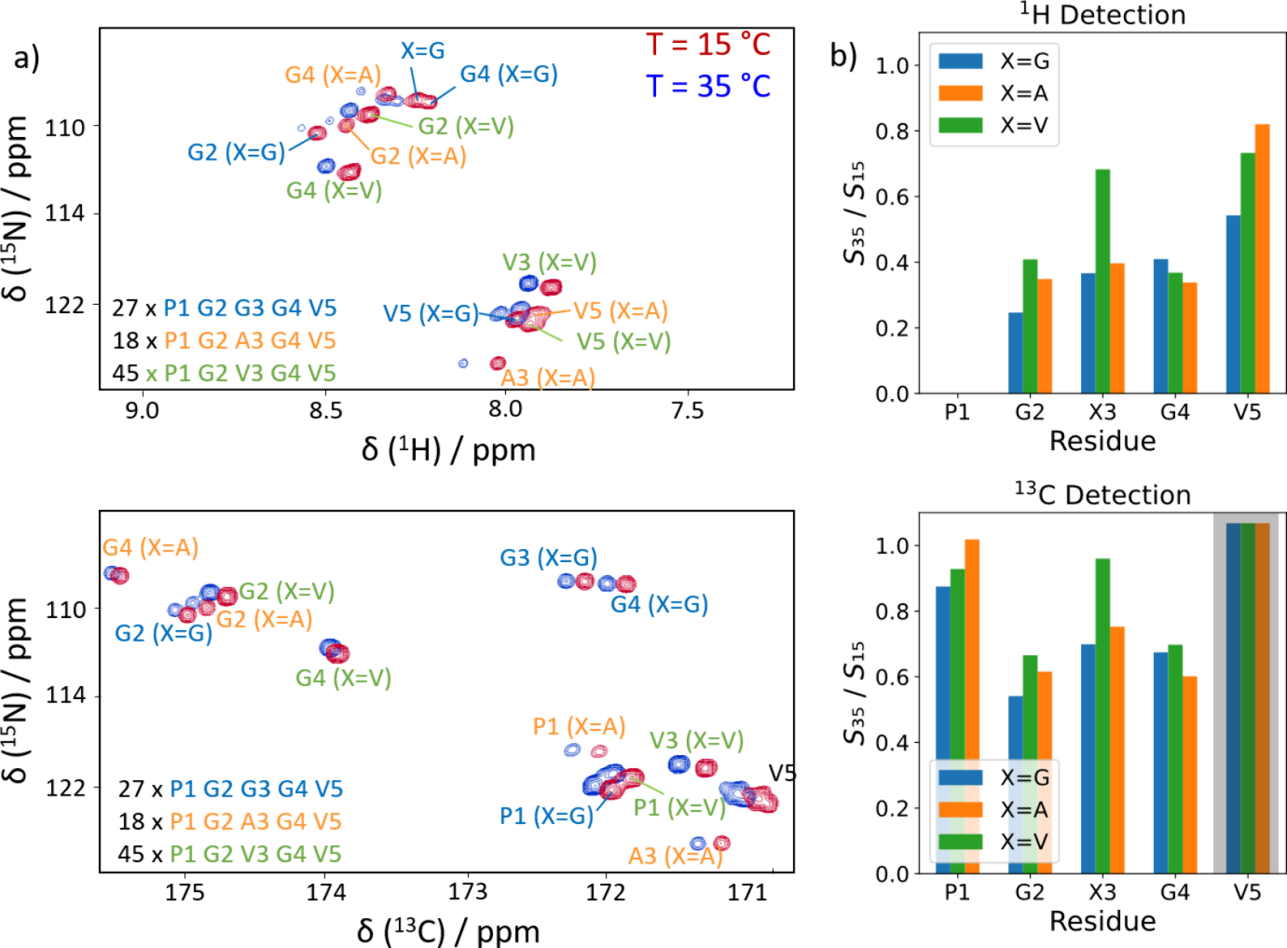
(a) ^1^H-^15^N and ^13^C-^15^N correlation spectra of the used ELP at 15 and 35 °C, i.e., below and above the LCST, respectively. The resonance assignment is indicated. (b) Signal amplitude ratios *S*_35_/*S*_15_ between the spectra recorded at 35 °C (*S*_35_) and 15 °C (*S*_15_). The signal amplitudes drop significantly more in the case of ^1^H detection upon heating. Note that the V5 signals (marked with the gray shade) overlap significantly at 35 °C using ^13^C-detection, such that the observed values cannot be interpreted reliably.

Figure 1a shows the ^1^H-^15^N HSQC (heteronuclear single quantum coherence) spectrum of uniformly isotope-enriched ELP below and above the LCST, as well as its ^13^C-^15^N counterpart. It should be noted that the signal intensity *S* in the former case significantly dropped as compared to the latter upon aggregation (Figure 1b). Using ^1^H-detection, even up to 80% of the original signal intensity is lost above the LCST. Evidently, as the ELP aggregates, the signals become broadened beyond the detection threshold.

Using the Relax-EXSY approach^49^ in combination with the herein-proposed detection scheme, it was possible to evaluate the proton exchange rates within the ELP aggregates. We found that at 35 °C the *k*_*ex*_ rate constant varied between 14 ± 2 and 16 ± 2 s^–1^ for most residues (Supporting Information Figure S2). These values indicate quite fast exchange, which can broaden resonances and, thus, explain why ^1^H-detected NMR only picked up the residual monomer species in solution but not the aggregates. However, it should be noted that this approach is only sensitive to exchange rates on the order of the time scale of longitudinal relaxation. Hence, even faster-exchanging species cannot be excluded. Further, the intrinsic hydrogen exchange rates (and thereby their effect on ^1^H-detected signal intensities) depend strongly on amino acid sequence.^57^

Nonetheless, this effect of proton exchange-driven line broadening is mitigated by the heteronuclear detection scheme. In this case, most of the signals retain their original intensities, notably the prolines that are important for the functioning of ELP (*vide infra*). The glycine residues get broadened but do not drop below the detection threshold (Figure 1). The signal intensities clearly show that ^13^C’-detection captures all ELP resonances even above the LCST (signal assignments and chemical shift perturbations are shown in Table S1 and Figure S3 together with a brief discussion). DOSY (diffusion-ordered spectroscopy) experiments further supported that ^13^C-detection effectively picked up the larger ELP aggregates (see the Supporting Information Figure S4 and Table S2).

To understand the dynamics of the system, we then recorded ^15^N-longitudinal *R*_1_ and ^15^N-transverse *R*_2_ relaxation rate constants using the conventional ^1^H^N^ amide-detection approach as well as the H^α^-CON-type pulse sequence detecting ^13^C’ nuclei developed by Felli, Pieratelli, and co-workers.^58−64^ Further, it should be noted that we recorded the relaxation rates with a CPMG interpulse duration of 1 < 2πJ such that the contribution to relaxation by antiphase coherences can be neglected.

The results are shown in Figure 2 (all raw data and fits are shown in Figures S3–S12). For the case of ^1^H^N^-detection, we found that relaxation rate constants were rather homogeneous.

**Figure 2:**
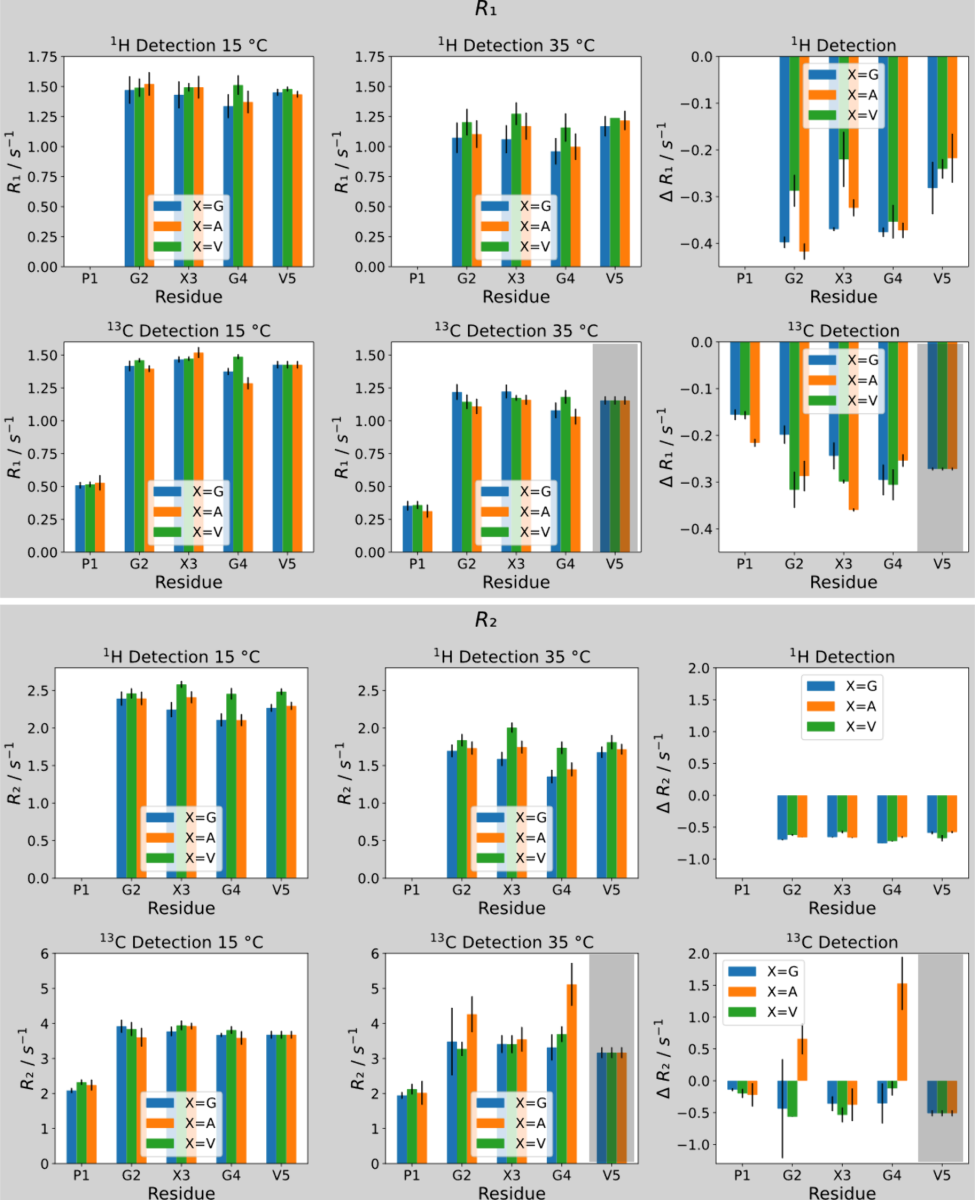
^1^H- as well as ^13^C-detected ^15^N longitudinal (*R*_1_) and transverse (*R*_2_) relaxation rate constants at 15 and 35 °C, as well as differential values Δ*R*_1_ and Δ*R*_2_, calculated as the difference between the high-temperature cases minus the low-temperature cases. Note that values highlighted with the gray shade stem from overlapping resonances and cannot be reliably interpreted. Note that the *y*-axis scale changes between the different panels.

**Figure 3:**
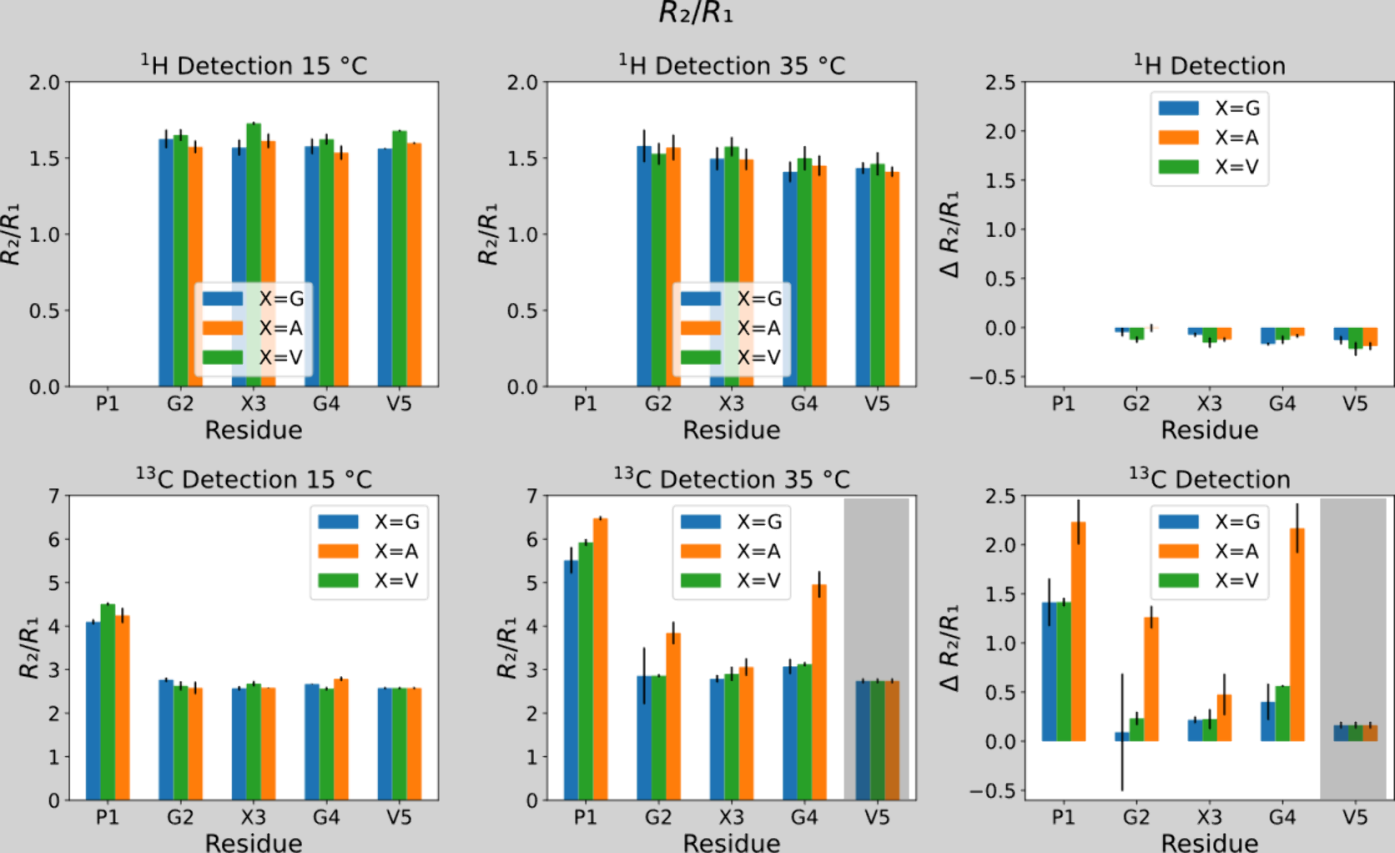
^1^H- as well as ^13^C-detected ^15^N *R*_2_/*R*_1_ ratios at 15 and 35 °C, as well as differential values Δ(*R*_2_/*R*_1_) and Δ*R*_2_, calculated as the difference between the high-temperature cases minus the low-temperature cases. Evidently, the behavior of the proline residues stands out, as do the glycines in the PGAGV repeats. Note that values highlighted with the gray shade stem from overlapping resonances and cannot be reliably interpreted.

*R*_1_ was ca. 1.4 s^–1^ for all resonances below the LCST and 1.0–1.2 s^–1^ above the LCST, *R*_2_ was 2.0–2.4 s^–1^ below the LCST and 1.4–1.8 s^–1^ above the LCST. Interestingly, the temperature-induced changes Δ*R*_2_ were very similar for all resonances, i.e., −0.6–0.7 s^–1^ in all cases in the sense that lower rates were observed at higher temperatures. In other words, at higher temperatures, less efficient transverse relaxation indicates higher mobility of the detected ELP species. This observation demonstrates that the conventional ^1^H-detection scheme recorded only the relaxation properties of the monomers, which remained in solution, as already suggested by the strong drop in signal intensity found in the 2D correlation spectrum (cf. Figure 1). The homogeneity of the recorded relaxation rates is well reflected in *R*_2_/*R*_1_ ratios. (It should be noted that the proportionality of R_2_/R_1_ ratios with rotational correlation times τ_c_ is only valid in the intermediate/slow-tumbling regime and under conditions of spherical rotation. Both conditions are not necessarily fulfilled for the present case. Hence, the ratios should be interpreted only as a relative measure of local residue mobility, but not in a quantitative fashion.^65^) For all residues the differential values Δ(*R*_2_/*R*_1_), i.e., the difference of the ratio below and above the LCST was between −0.0 and −0.2 (Figure 3). The *R*_2_/*R*_1_ ratio below and above the LCST was only 1.4–1.6 for all resonances (Figure 3), meaning that the resonances reported on a highly dynamic system, as typical for disordered peptides, and again pointing toward the fact that the conventional proton-based NMR approach is not sensitive to the signature of the ELP aggregates.^14,31^ Similar observations were already made earlier by Reichheld et al.^27^

It should be stressed again that the ^1^H-detected HSQCs in Figure 1b show reduced signal intensity upon aggregation as this detection scheme only records monomer species not assembled in ELP aggregates. Consequently, the relaxation rates recorded via the ^1^H-^15^N HSQC also always show only small relaxation rate differences.

A drastically different picture emerged when we employed the H^α^-CON-based pulse sequences, with which we could detect the ELP aggregates, in contrast to the ^1^H-detection-based scheme. The obtained ^15^N-relaxation rates clearly reflected the different dynamics within the ELP aggregates.

The *R*_1_ rates significantly dropped upon surpassing the LCST for all residues by 0.15–0.35 s^–1^, and the *R*_2_ values changed heterogeneously by −0.5 to +1.5 s^–1^ (Figure 2). Two further points stand out. First, the proline residues, which can now be detected,^66^ experience only very little change in *R*_2_ upon aggregation despite a rise in temperature of 20 °C, pointing toward compensation of the temperature effect. Second, glycine residues in PGAGV-type pentapeptide blocks experience a significantly higher *R*_2_ rate compared to glycine in PGGGV and PGVGV pentapeptide blocks (Figure 2).

To identify whether the observed effects are due to confined mobility or conformational exchange contributions to *R*_2._ We also recorded constant-time experiments at different CPMG frequencies ν_CPMG_ (see the Supporting Information Figures S13 and S14). The ELP resonances showed no dependence on ν_CPMG_, indicating that the changes in *R*_1_ and *R*_2_ are due to reduced mobility upon aggregation.

The effect is again best visualized through the *R*_2_/*R*_1_ ratios in Figure 3. The increased ratios clearly show how the prolines experience a strongly reduced rotational freedom that contributes to *R*_2_ upon aggregation. In contrast to most other residues, which showed only weaker responses upon heating the system above the LCST, only the glycine residues in PGAGV blocks display a similar increase in *R*_2_/*R*_1_.

The *R*_2_/*R*_1_ ratios for the proline and glycine residues rose (in contrast to the case of ^1^H^N^-detection) up to values of 6 upon aggregation, which indicates a rise in rotational correlation times.^67^ Such increasing *R*_2_/*R*_1_ ratios are a clear indication of ELP aggregation and the concomitant changes in local structural dynamics. The slower motions of the proline residues in the ELP aggregates point toward a strong involvement of these residues in the aggregation process. This observation is well in line with the earlier report of β-hairpin-type structures, which guide the hydrophobic proline side chain into the core of the aggregates,^26^ offering a contact interface for intermolecular assembly. Thus, our results agree with the interpretation by Li et al.^26^ that the proline residues indeed form the contact between different ELPs in the aggregates.

Due to the absence of any amide proton, the mechanisms inducing nuclear spin relaxation for prolines differ from those of other amino acids. As a result, the relaxation rate constants are smaller for prolines. Thus, the differential values Δ*R*_1/2_ are also smaller. Furthermore, the influence of chemical proton exchange on the adjacent ^15^N nucleus is canceled, such that Δ*R*_2_ values upon temperature shifts can become particularly small compared to other amino acids.

Interestingly, the ^13^C-derived *R*_2_/*R*_1_ ratios still report relatively fast motion within the ELP aggregates. Considering that an aggregate of only two ELP unimers already features a molecular weight of ∼40 kDa, a rotational correlation time of ≫10 ns would be expected for a rigid peptide structure.^68^ However, as we find *R*_2_/*R*_1_ ≈ 6 (corresponding to ca. 3 ns), a high degree of internal dynamics must necessarily be the underlying cause. Note that the UV data in Supporting Information Figure S1 shows that almost all ELP is aggregated under our experimental conditions. Hence, even in cases of exchange averaging between free and aggregated species, the above argument holds. Our data suggests that the interior of ELP aggregates, even quite hydrophobic ones as used herein, can be described as dynamic systems with high degrees of flexibility–reminiscent of condensed phases formed by intrinsically disordered proteins.^69−72^

**Figure 4:**
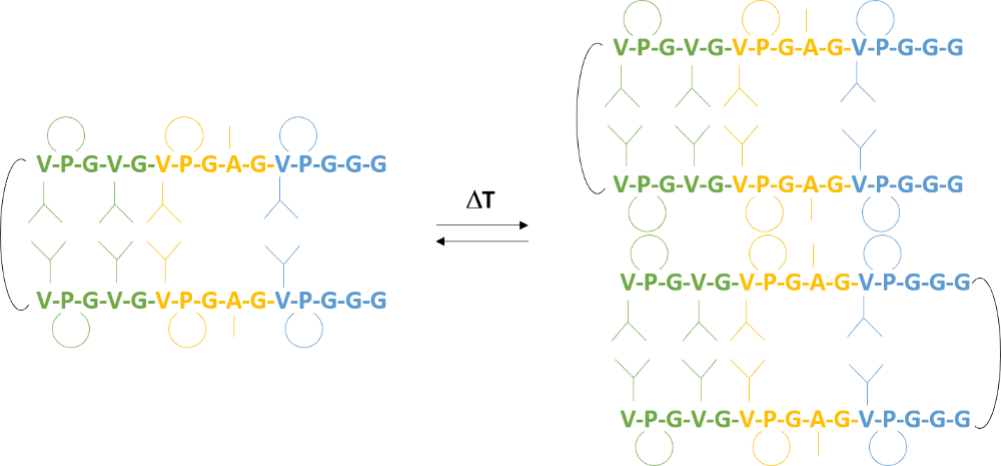
A coarse structural model that explains the reported relaxation rates. Structural elements are stabilized below the LCST via hydrophobic side chain contacts. Above the LCST proline residues are responsible for intermolecular attraction. This picture is supported by nuclear Overhauser spectroscopy (NOESY) data, which showed that the valine residues form a plethora of through-space contacts at 15 °C, i.e., far below the LCST (see the Supporting Information Figure S15), indicating a compacted state/fold.

Also important is the observation that the PGAGV blocks experience a much stronger reduced motional freedom than PGGGV and PGVGV blocks.^19,21,73^ It is well established that an increasing fraction of valine guest residues, as opposed to alanine guest residues, reduces ELP LCST. Hence, one would expect to observe a stronger involvement of PGVGV blocks in the aggregation process. While we do observe that these blocks are more involved than the PGGGV and PGCGV blocks, the prominent behavior of the alanine residues is nonetheless surprising. However, considering the reported formation of β-turn and helix structures already below the LCST^26^ and the involvement of valine guest residues in the stabilization of these structures, a possible, straightforward explanation for our observation is as follows: The pentapeptide blocks with valine guest residues are already involved in the intramolecular stabilization of structural interfaces via hydrophobic side chain contacts below the LCST. Above the LCST, the pentapeptide blocks with alanine guest residues, therefore, become more involved in the formation of new, intermolecular hydrophobic interfaces (Figure 4), leading to additional motional restrictions or contributions of conformational exchange to the transverse relaxation rate constants.

Finally, several points need to be considered concerning the methodology reported herein. First, the ELP aggregates may feature a broader molecular weight distribution, such that the ^13^C-detected spectra only record the lighter share of aggregates. Although typically ELP aggregates are monodisperse, this possibility cannot be excluded *a priori*. Second, the NMR experiments display only a single peak above the LCST, such that the ELP system appears to feature a fast exchange between monomers in solution and molecules in ELP aggregates. However, other explanations cannot be discarded a priori, such as dark states in slow exchange with a visible state. Third, it should be stressed that ELP are very favorable target molecules for the proposed methodology due to their low primary sequence heterogeneity. The pentapeptide repeat structure causes a large set of resonances to constructively superpose, such that the intrinsic signal strength is very high compared, e.g., to an intrinsically disordered protein with similar molecular weight.

## Conclusions

We report an NMR methodology that enables insights into the structural dynamics of large aggregates of genetically encoded polymers such as ELP. It allows for the characterization of the internal structural dynamics of these aggregates. A coarse-grained structural model reconcilable with the reported relaxation parameters is shown in Figure 4. In particular, ^13^C-detected ^15^N-*R*_2_/*R*_1_ ratios can help to identify differential dynamics within aggregates of ELP and derive this picture in which proline residues are key to the intermolecular hydrophobic contact interface behind the LCST transitions. Surprisingly, pentapeptide blocks with alanine guest residues display a peculiar behavior, i.e., significantly stronger impacted dynamics than glycine- or valine-housing pentapeptide blocks, possibly due to the latter’s involvement in intramolecular contacts even below the LCST. The presented study is the first proof of concept that ^13^C-direct detection is a viable tool to delineate the internal configuration of ELP aggregates by NMR. An extension of the ^13^C-^15^N-detection scheme to methods such as saturation transfer difference NMR to map contact interfaces or determining secondary structure content within the ELP aggregates can now be undertaken.

The presented approach might also be beneficial in the context of liquid–liquid phase separation of intrinsically disordered proteins, a topic that currently raises much interest. ^13^C-detected relaxation rates might help obtain a deeper look into the structural dynamics of condensed phases. Interestingly, we found that the reason for the failure of proton-detected NMR in resolving the internal structural dynamics of ELP is not due to slow molecular tumbling but rather due to exchange processes involving the amide H^N^ proton. Indeed, the herein-reported fast internal dynamics of ELP aggregates are reminiscent of the dynamics of condensed phases of intrinsically disordered proteins. Hence, on the one hand, the reported relaxation rates allow for the analysis of dynamics within a condensed liquid-like protein phase. On the other hand, the data suggests that ELP aggregates might be a simple model system for such intriguing phenomena.

## Supporting Information Available

ELP sequence, UV–vis data, relaxation data and fits, and CPMG data .(PDF)
