# Supplementary material for: The Internal Structural Dynamics of Elastin-Like Polypeptide Assemblies by 13C-Direct Detected NMR Spectroscopy
Source: Anal Chem. 2025 Feb 17;97(7):3937–44. doi: 10.1021/acs.analchem.4c05163 (PMC11866286; doi:10.1021/acs.analchem.4c05163)
Supplement: Supplementary file 1 — ac4c05163_si_001.pdf [file ac4c05163_si_001.pdf]

<u>**Supporting Information**</u>

The Internal Structural Dynamics of Elastin-Like Polypeptide Assemblies by ^{13}C-direct detected NMR Spectroscopy

Dörte Brandis[1,2], Pavel Kadeřávek[1,3,4], Dennis Kurzbach[1]*

[1]Institute of Biological Chemistry, Faculty of Chemistry, University of Vienna, Währinger Str. 38, 1090 Vienna, Austria
[2]University of Vienna, Vienna Doctoral School in Chemistry (DoSChem), Währinger Str. 42, 1090 Vienna, Austria
[3]Central European Institute of Technology (CEITEC), Masaryk University, Kamenice 5, 625 00 Brno, Czech Republic.
[4]National Centre for Biomolecular Research (NCBR), Faculty of Science, Masaryk University, Kamenice 5, 625 00 Brno, Czech

**E-Mail: dennis.kurzbach@univie.ac.at*

Complete protein sequence 2
Turbidity measurement 2
Relax-EXSY Data 3
NMR assignment 3
Chemical shift perturbations 4
Fits and results of ^{1}H and ^{13}C DOSY experiments 5
Supplementary material of 1H and 13C detected relaxation measurements 6
Experiments with different CPMG Frequencies 10
NOESY spectrum of ELP 11

Complete protein sequence

VGVPGVGVPGGGVPGAGVPGVGVPGVGVPGVGVPGGGVPGAGVPGGGVPGVGVPGVGVPGGGVP
GAGVPGVGVPGVGVPGVGVPGGGVPGAGVPGGGVPGVGVPGVGVPGGGVPGAGVPGVGVPGVGV
PGVGVPGGGVPGAGVPGGGVPGVGVPGVGVPGGGVPGAGVPGVGVPGVGVPGVGVPGGGVPGAG
VPGGGVPGVGVPGVGVPGGGVPGAGVPGVGVPGVGVPGVGVPGGGVPGAGVPGGGVPGVGVPGV
GVPGGGVPGAGVPGVGVPGVGVPGVGVPGGGVPGAGVPGGGVPGVGVPGVGVPGGGVPGAGVPG
VGVPGVGVPGVGVPGGGVPGAGVPGGGVPGVGVPGVGVPGGGVPGAGVPGVGVPGVGVPGVGVP
GGGVPGAGVPGGGVPGVGVPGVGVPGGGVPGAGVPGVGVPGVGVPGVGVPGGGVPGAGVPGGGV
PGENLYFQGSSKKSGSYSGSKGSKRRIL

Turbidity measurement

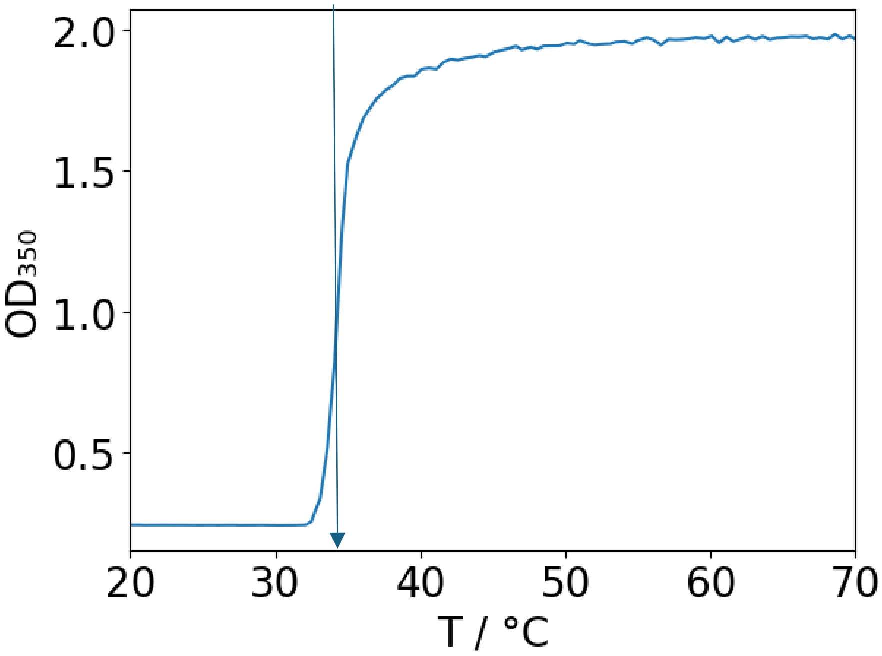

Figure S1. Optical density of the used ELP at the same conditions as the NMR measurements samples at a wavelength of 350 nm is dependent on the sample temperature. A temperature of 35° C (marked by the arrow in the Figure) corresponds to the best compromise between NMR detection and sample temperature above the LCST, which is <34° C.

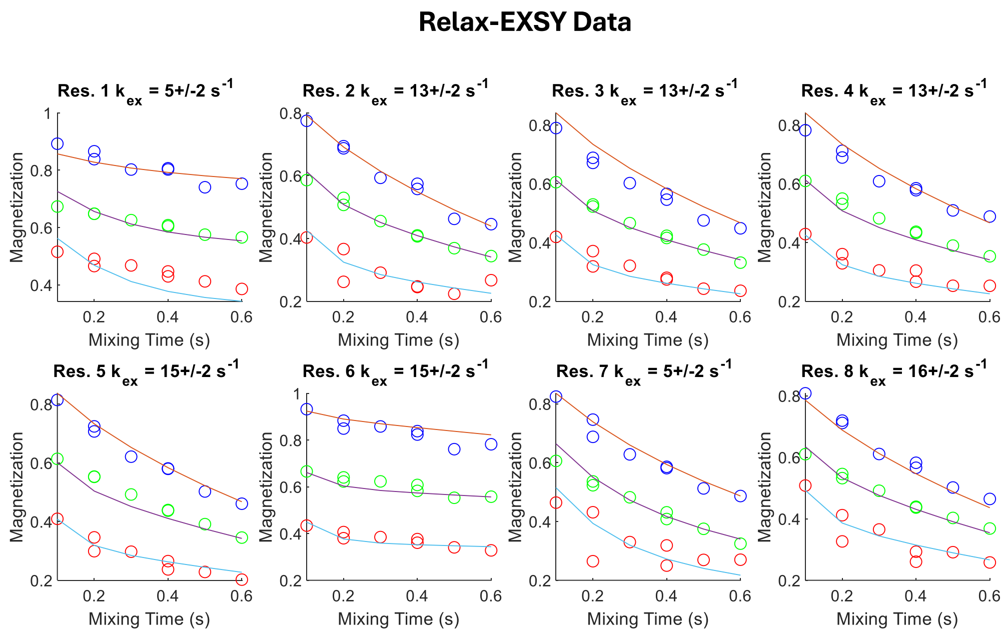

Figure S2. Exchange rates and fits to Relax-EXSY data. Blue green and circles indicate 9%, 30% and 50% D_2O content in the buffer. Solid lines correspond to fits to the data. In contrast, at 15° C, no dependence on the D2O concentration could be found, indicating a very slow chemical proton exchange.

NMR assignment

Table S1. Assignments for ^{13}C ^{15}N labeled ELP. The assignment was achieved by combining of HNCO, HNCACB, HNN and HSQC spectra.

	^{13}C-CO / ppm	^{15}N / ppm	^{1}H / ppm
**P1 (X=G)**	172.096	120.001	
**G2 (X=G)**	175.074	110.281	8.479
**G3 (X=G)**	172.301	108.405	8.195
**G4 (X=G)**	172.007	108.523	8.161
**V5 (X=G)**	171.081	120.790	7.938
**P1 (X=A)**	172.250	117.969	
**G2 (X=A)**	174.949	109.851	8.276
**A3 (X=A)**	171.368	123.251	7.979
**G4 (X=A)**	175.530	108.132	8.276
**V5 (X=A)**	171.081	120.555	7.898
**P1 (X=V)**	171.963	119.376	
**G2 (X=V)**	174.801	109.265	8.330
**V3 (X=V)**	171.500	119.110	7.844
**G4 (X=V)**	173.971	112.625	8.384
**V5 (X=V)**	171.081	121.141	7.911

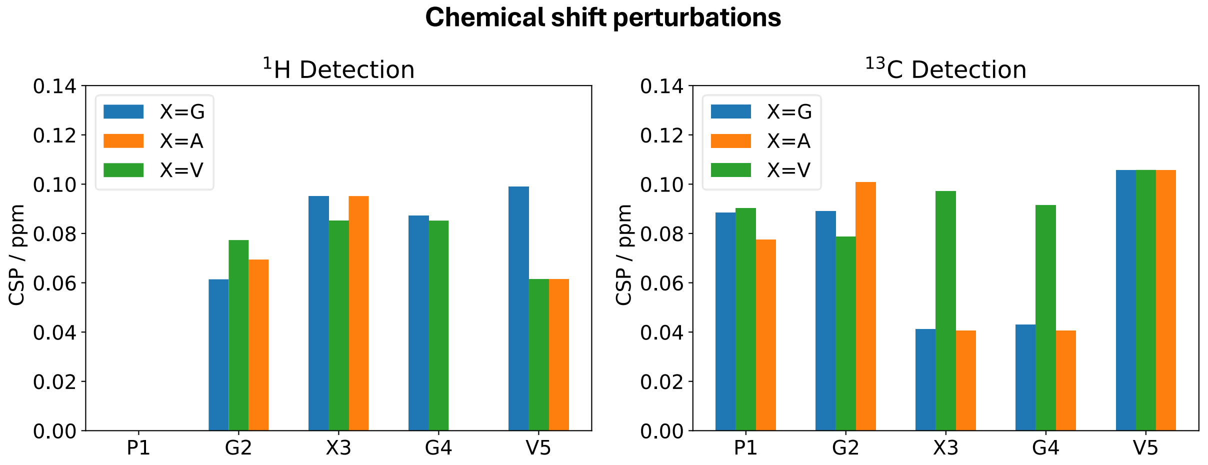

Figure S3. Chemical shift perturbation of ^{1}H and ^{13}C - detected relaxation measurements of the ELP upon increasing the sample temperature from 15 to 35 °C inducing ELP aggregation. Note that the chemical shift differences between 15 and 35° C are minor with ^{1}H as well as with ^{13}C detection. Assuming the absence of substantial changes in the time-averaged conformations and their H-bond interactions, this suggests that the system is in the fast exchange between condensed and monomeric phases. The differential weighting between ^{1}H and ^{13}C' detected methods between monomeric and phase-separated states might, thus, cause the observed different chemical shift perturbations.

Fits and results of ^1H and ^{13}C DOSY experiments

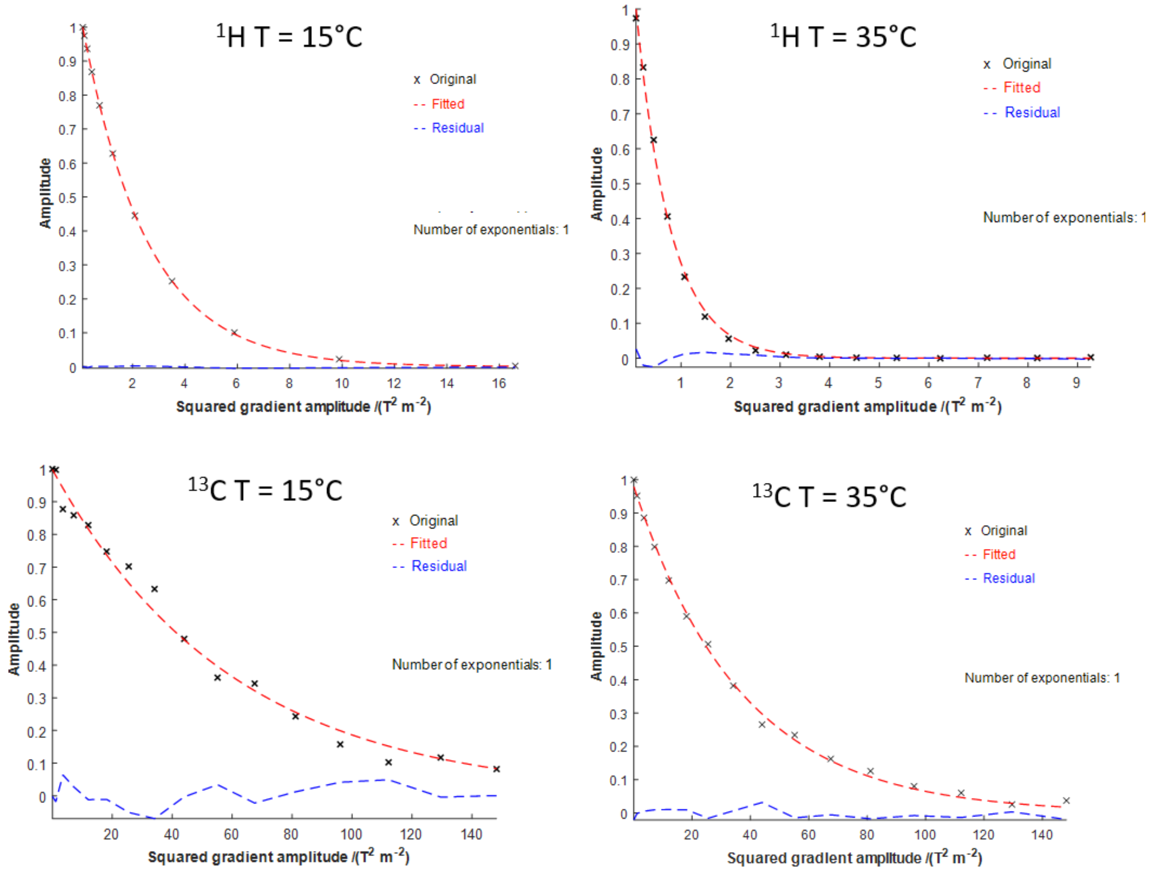

Figure S4. DOSY data and fitting at 15 °C (left) and 35 °C (right) were obtained with a ^1H-Dosy and ^{13}C DOSY. For ^{13}C DOSY the data was smoothed using a moving average of two. 1,4-Dioxane was used for internal referencing.

Table S2. Obtained diffusion coefficients of fits, shown in Figure S12, and calculated radius.

Nucleus	T / °C	r / nm
^1H	15	2.1+/-0.1
^1H	35	2.1+/-0.1
^{13}C	15	2.3+/-0.3
^{13}C	35	3.8+/-0.2

Supplementary material of 1H and 13C detected relaxation measurements

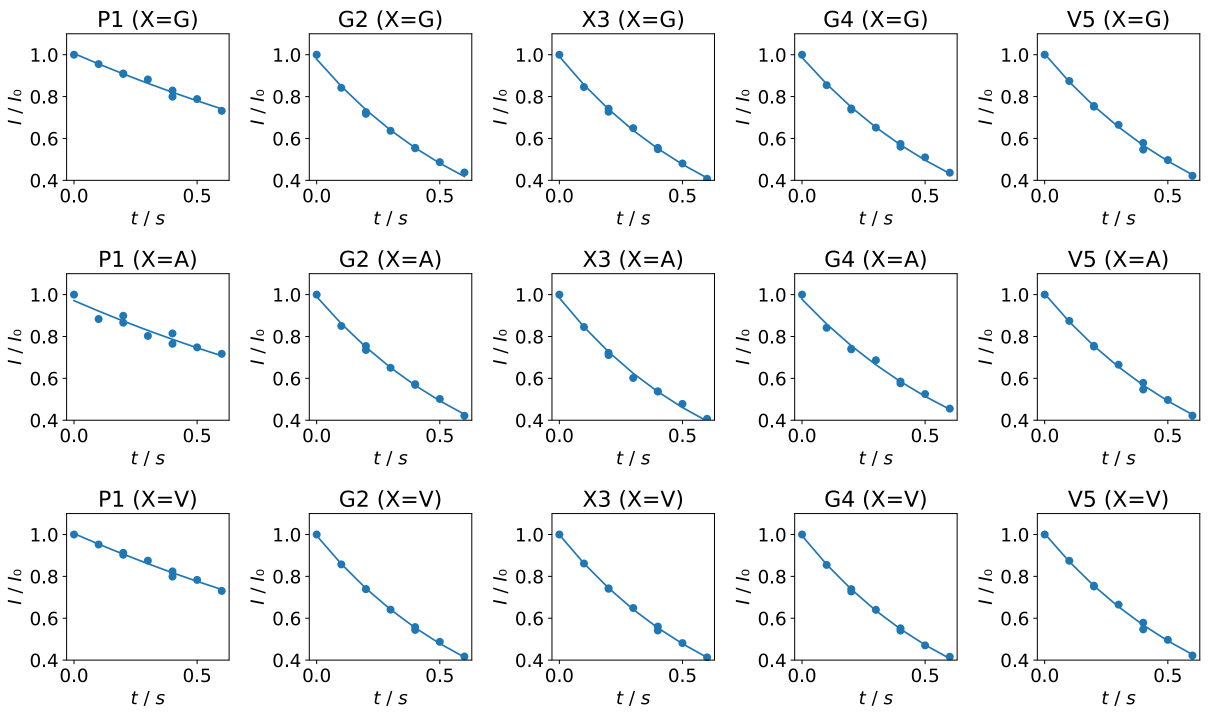

Figure S5. Relaxation fits of ^{13}C - detected R_1 measurements at 15 °C.

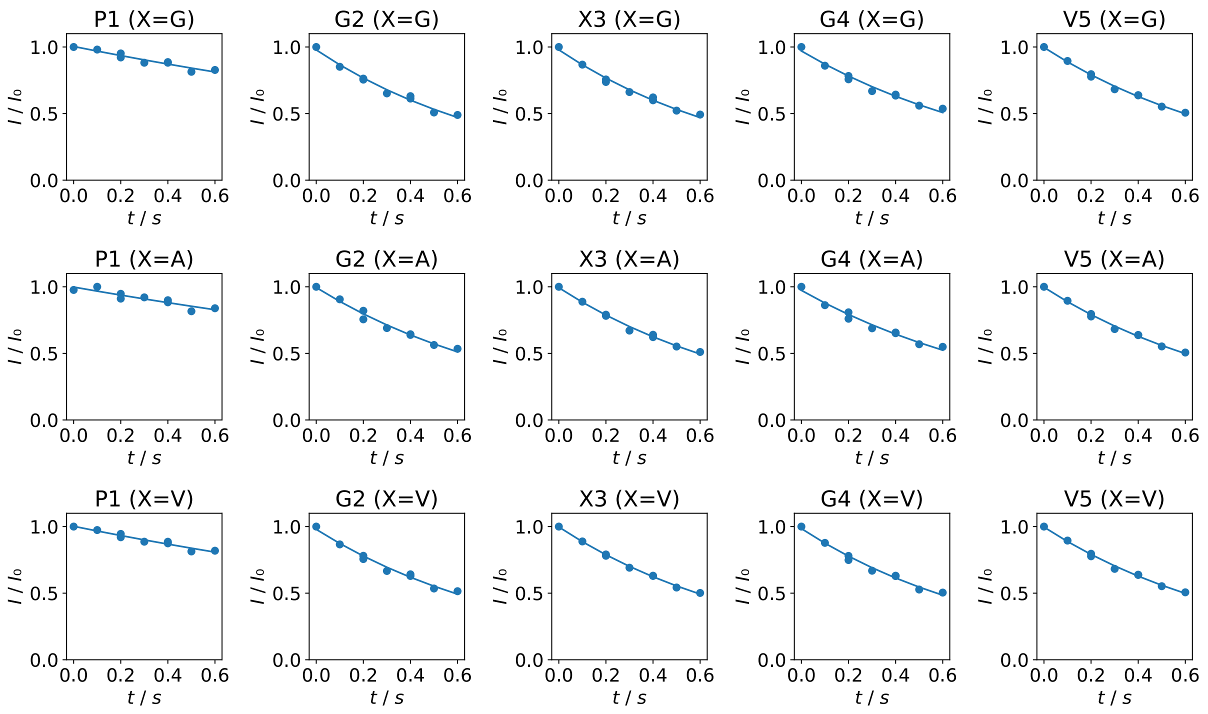

Figure S6. Relaxation fits of ^{13}C - detected R_1 measurements at 35 °C.

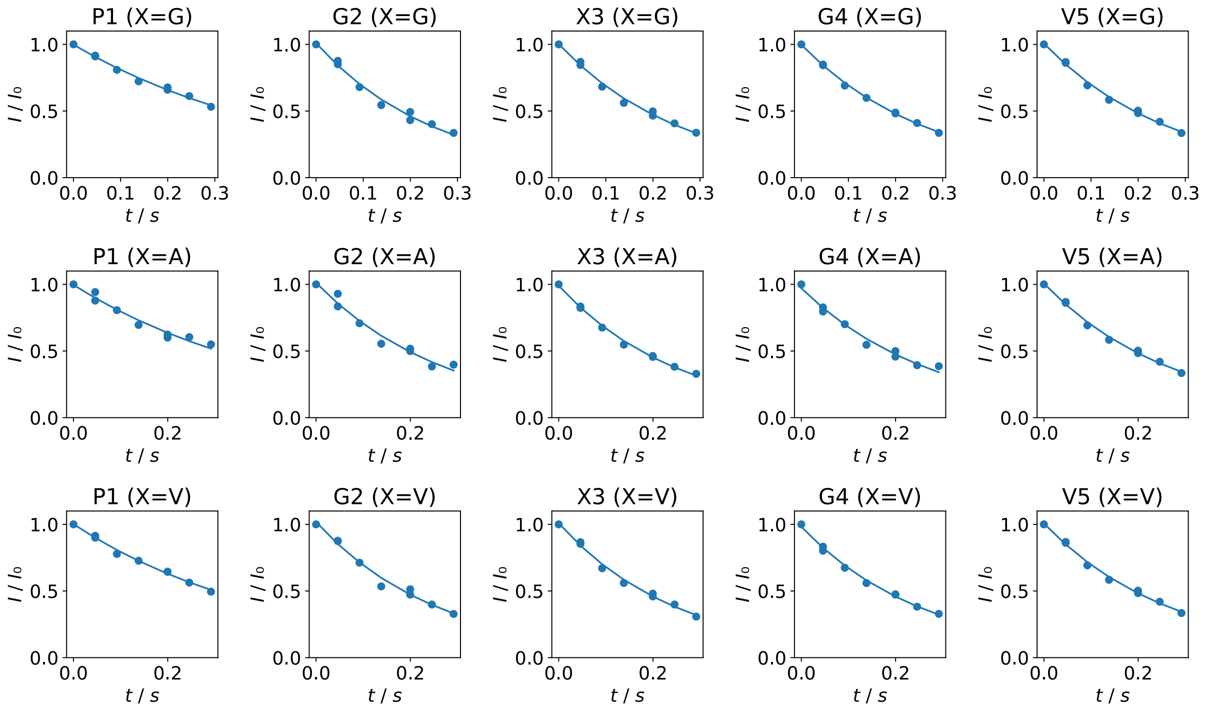

Figure S7. Relaxation fits of [13]C - detected R_2 measurements at 15 °C.

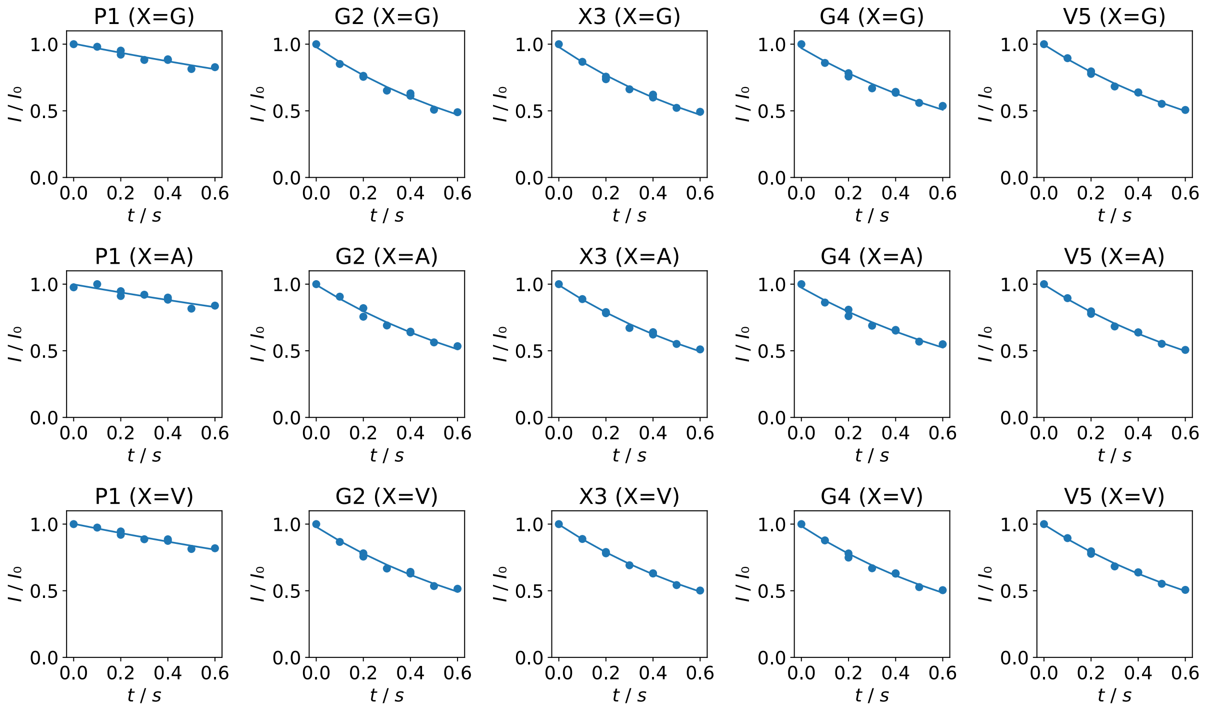

Figure S8. Relaxation fits of [13]C - detected R_2 measurements at 35 °C.

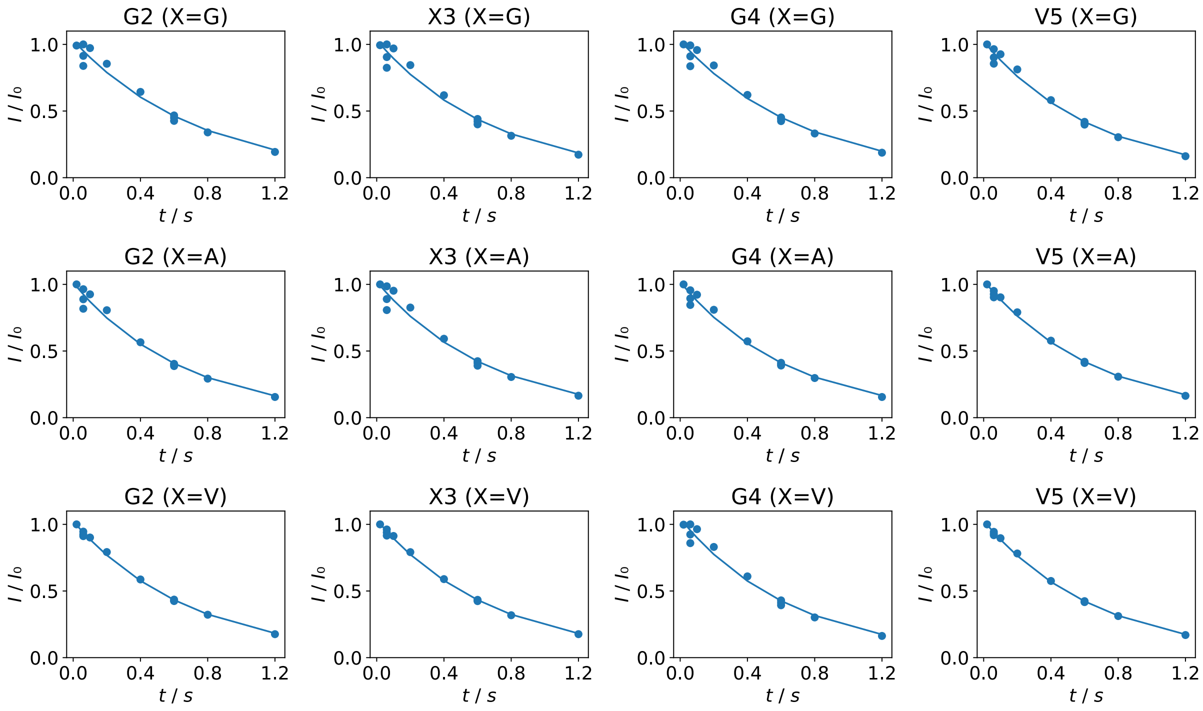

Figure S9. Relaxation fits of [1]H - detected R_1 measurements at 15 °C.

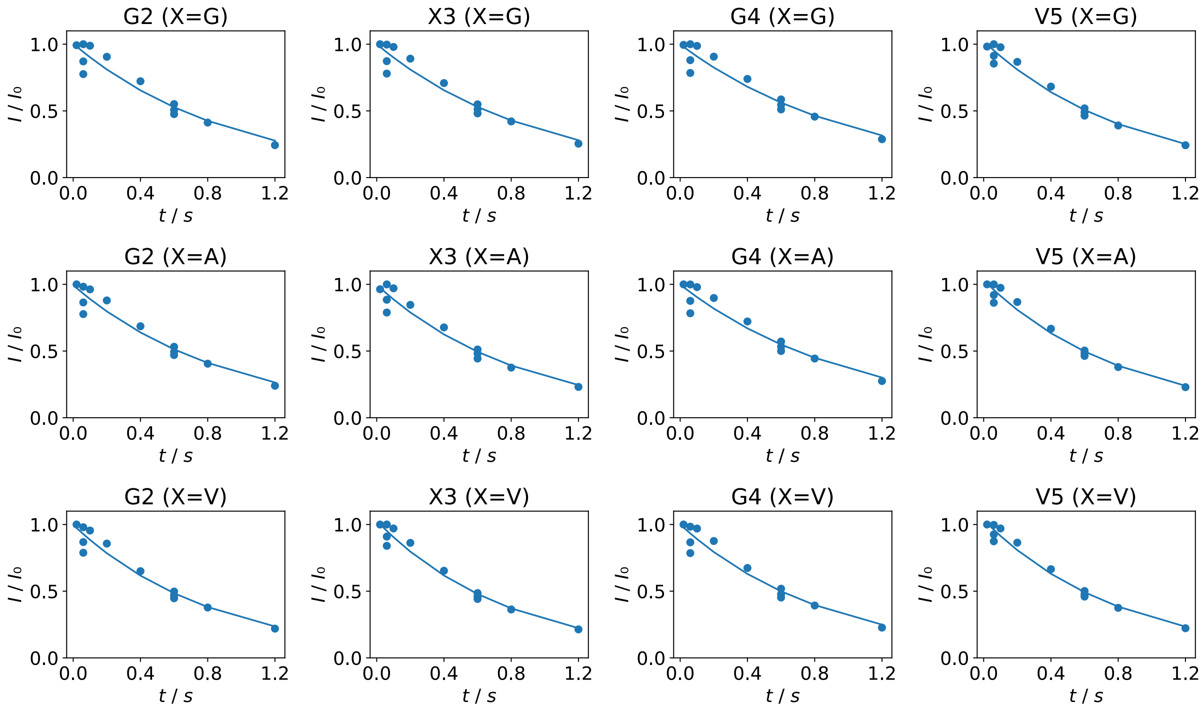

Figure S10. Relaxation fits of [1]H - detected R_1 measurements at 35 °C.

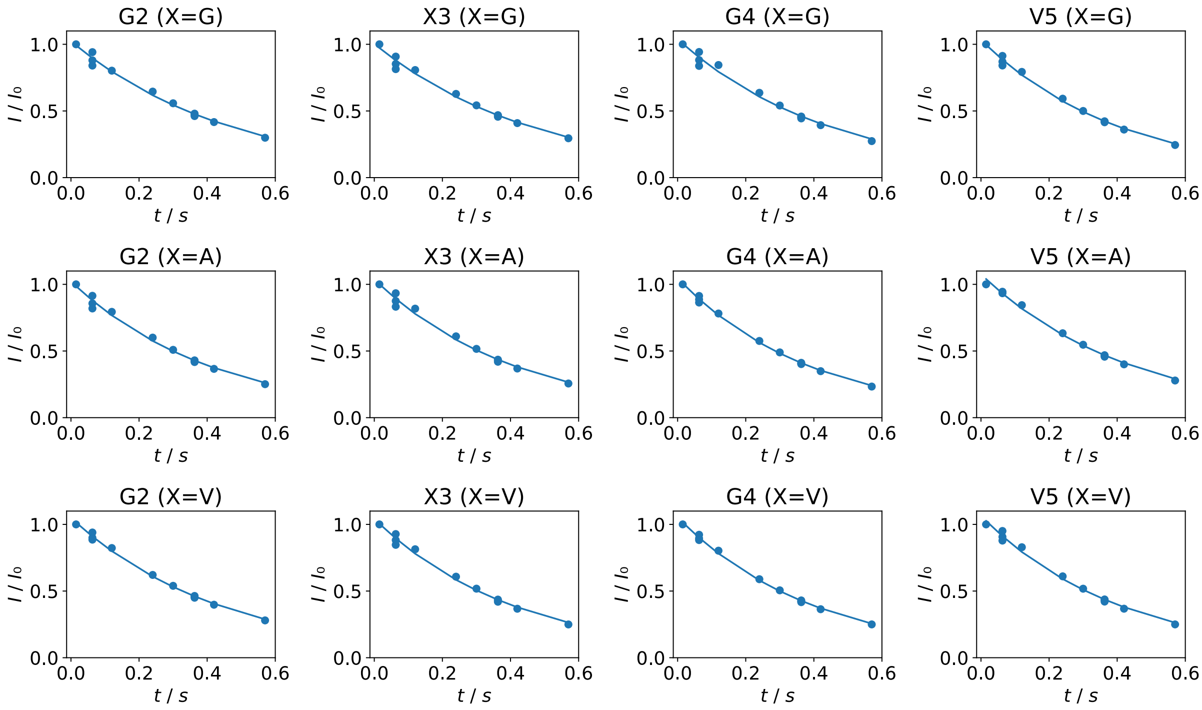

Figure S11. Relaxation fits of [1]H - detected R_2 measurements at 15 °C.

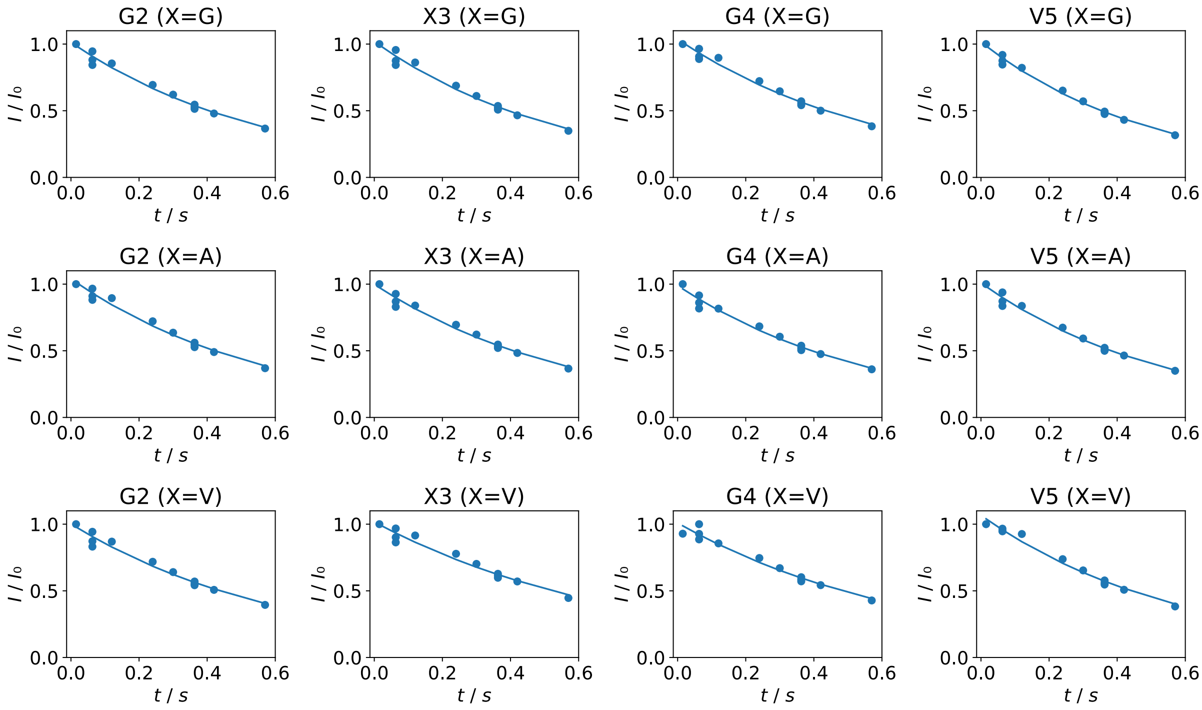

Figure S12. Relaxation fits of [1]H - detected R_2 measurements at 35 °C.

Experiments with different CPMG Frequencies

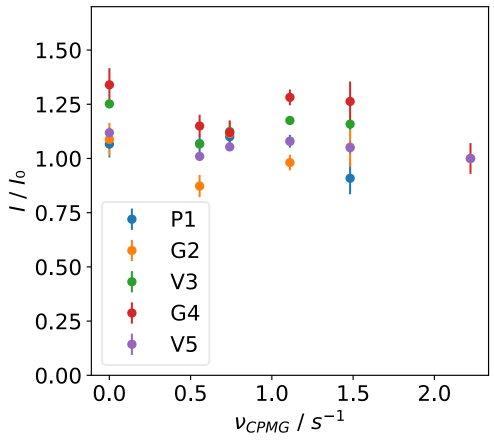

Figure S13. The normalized of ^{13}C - detected signal intensity R_2 at 35 °C depending on the CPMG frequency with a total duration of the CPMG block of 0.05 s.

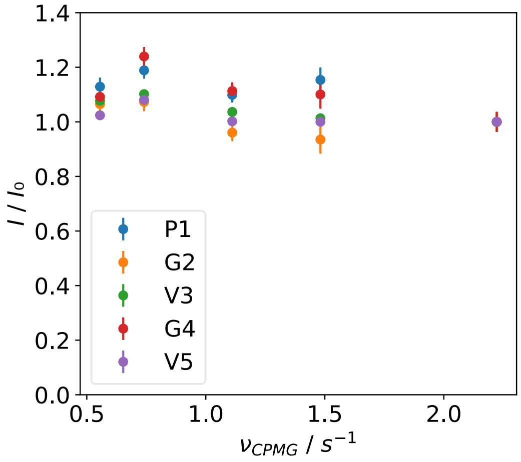

Figure S14. The normalized of ^{13}C - detected signal intensity R_2 at 35 °C depending on the CPMG frequency with a total duration of the CPMG block of 0.1 s.

NOESY spectrum of ELP

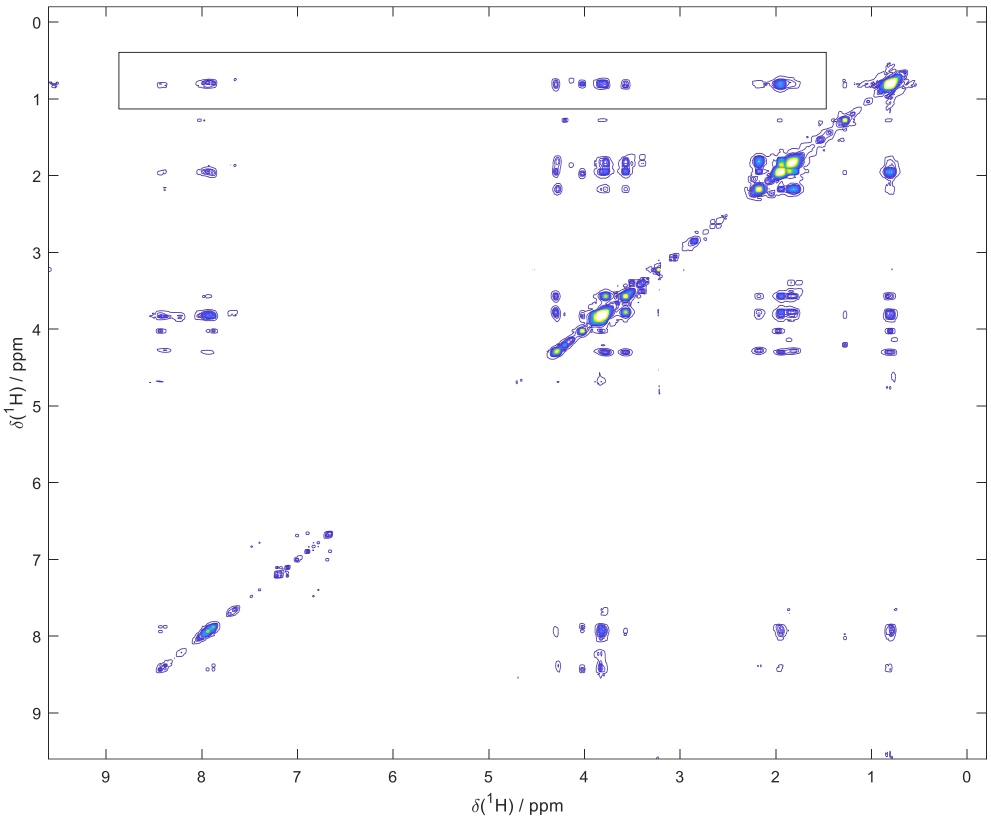

Figure S15. NOESY, recorded with a mixing time of 300 ms, of the studied ELP recorded at 15° C. Cross peaks between the valine methyl resonances and several HN as well Hα resonances (indicated by the black box)support the idea that the ELP is already compacted at temperatures below the LCST
